# Acute Sirolimus Overdose: A Multicenter Case Series

**DOI:** 10.1371/journal.pone.0128033

**Published:** 2015-05-28

**Authors:** Alessandro Ceschi, Elja Heistermann, Sonja Gros, Cornelia Reichert, Hugo Kupferschmidt, Nicholas R. Banner, Stephan Krähenbühl, Anne B. Taegtmeyer

**Affiliations:** 1 Swiss Toxicological Information Centre, Associated Institute of the University of Zurich, Zurich, Switzerland; 2 Department of Clinical Pharmacology and Toxicology, University Hospital Zurich, Zurich, Switzerland; 3 Berlin Poison Information Centre, Berlin, Germany; 4 Mainz Poison Control Centre, Mainz, Germany; 5 The Royal Brompton and Harefield NHS Foundation Trust, Harefield Hospital, Harefield, Middlesex, United Kingdom; 6 National Heart and Lung Institute and Institute of Cardiovascular Medicine and Research, Imperial College, London, United Kingdom; 7 Department of Clinical Pharmacology and Toxicology, University and University Hospital Basel, Basel, Switzerland; University of Toledo, UNITED STATES

## Abstract

**Background:**

There are few data relating to sirolimus overdose in the medical literature. Our objectives were to describe all cases of overdose with sirolimus reported to Swiss, German and Austrian Poisons Centres between 2002-2013.

**Methods:**

An observational case-series analysis was performed to determine circumstances, magnitude, management and outcome of sirolimus overdose.

**Results:**

Five cases of acute sirolimus overdose were reported – three in young children and two in adults. Four were accidental and one was with suicidal intent. Two patients developed symptoms probably related to sirolimus overdose: mild elevation of alkaline phosphatase, fever and gastroenteritis in a 2.5-year-old male who ingested 3 mg, and mild changes in total cholesterol in an 18-year-old female after ingestion of 103 mg. None of these events were life-threatening. Serial blood concentration measurements were performed starting 24 h after ingestion of 103 mg in a single case, and these followed a similar pharmacokinetic time-course to measurements taken after dosing in the therapeutic range.

**Conclusions:**

Acute sirolimus overdose occurred accidentally in the majority of cases. Even large overdoses appeared to be well-tolerated, however children might be at greater risk of developing complications. Further study of sirolimus overdose is needed.

## Introduction

Sirolimus (formerly rapamycin) is an immunosuppressive agent licensed for the prophylaxis of organ rejection in renal transplant recipients [1]. It is recommended that sirolimus be used initially in combination with cyclosporine and corticosteroids, with subsequent withdrawal of cyclosporine as necessary [1]. It is also used off-label in other types of organ transplant. Sirolimus inhibits activation and proliferation of T lymphocytes and antibody production by a distinct mechanism. In cells, it binds to the immunophilin, FK Binding Protein-12 (FKBP-12). The resulting complex in turn inhibits the activation of a key regulatory kinase, mammalian target of rapamycin (mTOR). Failure to activate mTOR results in inhibition of Interleukin-2-driven T-cell proliferation [2].

Sirolimus is a cytochrome P450 3A4 and P-glycoprotein substrate, making it susceptible to interactions with drugs that induce or inhibit the activity of these drug-handling proteins [1]. The maximum licensed starting dose of oral sirolimus in combination with cyclosporine (a cytochrome P450 3A4 and P-glycoprotein inhibitor) for adults and children over 13 years in Switzerland is 6 mg/day [1]. Absorption and metabolism of sirolimus is highly variable [3], and this results in great differences in blood concentrations between patients who have received the same dose. Subsequent dosing is therefore guided according to trough whole blood concentrations. A target trough concentration range of 5–15 μg/L when sirolimus is used concomitantly with cyclosporine and prednisone has been shown to be associated with effective rejection prophylaxis with minimal concentration-dependent adverse effects (leucopenia, thrombocytopenia, and hypertriglyceridemia) [3]. In the absence of cyclosporine, a four-fold increase in dose is usually required, however it is recommended that the daily dose should not exceed 40 mg [1]. Hypercholesterolaemia is a commonly reported adverse effect of sirolimus [1], however it appears to be only weakly dose-related [3].

Patients taking sirolimus or their household contacts who could have access to their drugs may be exposed to overdose, however, little is known about the effects of sirolimus in acute overdose. Sirolimus has a long half-life (62 ± 12 h in a study of renal transplant patients receiving concomitant cyclosporine and prednisone [4]), therfore a single overdose may have a prolonged effect. One source briefly mentions two cases of overdose with sirolimus [5]. In the first case, ingestion of 120 mg sirolimus was well tolerated, and in the second case atrial fibrillation developed after ingestion of 150 mg. A dose-escalation study in renal transplant patients taking cyclosporine examined the effect of single doses up to 15 mg/m^2^ [5, 6]. The only safety issues which arose were a case of transient thrombocytopenia (probably related to the single 28 mg sirolimus dose) and a case of mild epistaxis (possibly related to the single 21 mg sirolimus dose). A further study administered single doses of 21 mg/m^2^ to three stable renal transplant patients receiving cyclosporine [7], none of whom experienced toxicity. All this information relates to adults. There are no reports in the literature of sirolimus overdose during the post-marketing period.

The purpose of this study was to investigate the circumstances, management and outcomes of overdoses with sirolimus in adults and children using data reported to Swiss, German, and Austrian poisons centres.

## Materials and Methods

### Study design and inclusion criteria

A specific ethics approval was not required for this observational study due to the nature of the study design according to the regulations of the cantonal ethics committee Zurich, Switzerland at the time when the study was performed which also stated that anonymised data generated during patient care can be used retrospectively for research purposes without obtaining written consent. The clinical toxicologist who first advised on a case assigned it an internal unique case identifier. Once all data pertaining to the case (including follow up) had been obtained, the case was closed and the data—excluding patient-identifying information—was exported to a research database. This research database to which the authors had access therefore did not contain any patient-identifying information.

We performed a multicentre retrospective review of all acute overdoses involving sirolimus in adults and children (<16 years) either alone or in combination with other drugs that have been reported anonymously according to the Codex of the Society of Clinical Toxicology (GfKT) [8] to Swiss (Zurich), German (Berlin, Bonn, Erfurt, Freiburg, Göttingen, Mainz, Munich) and Austrian (Vienna) poisons centres between January 2002 and December 2013. The data, entered by physicians, included age, sex, weight, ingested drug and dose, symptoms/signs/laboratory values and causal relationship, severity of intoxication, decontamination measures, latency to decontamination, and therapeutic interventions.

### Circumstances and symptoms of overdose

The circumstances of overdose were categorised as `suicidal`for cases of intentional overdose, `domestic`for cases of accidental overdose in the home (primarily in children) and `iatrogenic`for those due to a prescribing or administration error in hospital.

The severity of symptoms were graded in accordance with the Poisoning Severity Score (PSS) as ‘minor’, for mild, transient and spontaneously resolving symptoms/signs; ‘moderate’, if at least one pronounced or prolonged symptom/sign was recorded; ‘severe’, if at least one severe or life-threatening symptom/sign was observed, or `fatal`, if the overdose was the recorded cause of death [9].

Cases were assessed for association between symptoms or signs and the immunosuppressant overdose by an expert panel including a senior clinical pharmacologist and a senior clinical toxicologist, both with additional qualifications in general internal medicine, using the World Health Organisation Uppsala Monitoring Centre (WHO-UMC) standardised case causality assessment criteria originally developed for the assessment of adverse drug reactions [10]. Comorbidities, co-ingestion of other medication (in patients with multiple drug overdose or taking other drugs in the therapeutic dose range) and the magnitude of overdose were taken into consideration. Associations were classified as `certain`, `likely`, `possible`and `unlikely`(Table A in S1 File).

### Statistical analyses

Overdoses in mg/kg were compared with usual therapeutic doses by determining the multiple of the subject`s usual therapeutic dose (dose received/usual dose) as performed in our previous studies [11–13]. Sirolimus is not licensed for children under the age of 13 years, so no calculation of the extent of overdose could be made. Missing data regarding patient weight was computed as detailed in Section A of the S1 File.

### Pharmacokinetic calculations

Half-life, clearance/bioavailability and apparent volume of distribution were calculated according to the standard pharmacokinetic equations given in Section A of the S1 File and PKSolver for Microsoft Excel was used for graphic representation [14].

## Results

There were a total of 367,445 reports to the Swiss Toxicological Information Centre of confirmed or suspected overdose with any substance by healthcare professionals during the study period. Of these, three were with sirolimus (Table 1 patients 1–3). Two cases were reported to German poisons centres during the same time period (Table 1 patients 4 and 5). There were no reports to the Austrian poisons centre.

**Table 1:** Patient demographics, circumstances of overdose and overdose amount.

Patient	Age (years)	Sex	Weight (kg)	Underlying condition	Circumstance of overdose	Mono Intoxication	Formulation	Dose (mg)	Dose (mg/kg)	Subject's usual dose (mg/d)
1	2.5	m	13	None	Domestic	Y	tablets	3	0.23	0
2	3	f	15	None	Domestic	N	tablets	2	0.13	0
3	18	f	55.8^a^	Liver transplantation (Wilson`s disease)	Suicidal	Y	tablets	103	1.85	1.5
4	1.9	m	13	None	Domestic	Y	tablets	max. 7	max. 0.54	0
5	58	m	84	Renal transplantation	Iatrogenic	Y	oral suspension	1.1 (equivalent to 6.7 mg oral^b^)	0.08	uk

Abbreviations: f = female, i.v. = intravenous, m = male, max. = maximum, N = no, uk = unknown, Y = yes.

^a^weight not available so mean weight given (see Section A of the S1 File)

^b^assuming concomitant ciclosporin therapy).

### Circumstance of overdose

Tables 1 and 2 show the subjects’ characteristics and the circumstances of overdose. Three of the five cases (60%) involved accidental overdose in young children in the home-setting, one case of overdose was with suicidal intent and one involved an iatrogenic administration error.

**Table 2:** Overdosage as a multiple of the patient’s usual dose (or factor above maximum licensed dose), decontamination measures and clinical findings.

Patient	Multiple of usual or maximum licensed dose	Management	Clinical findings (within number of days after overdose)	Severity	Relatedness to overdose
1	n/a	Single dose charcoal 1g/kg	Increased alkaline phosphatase (<2-fold) (2)	Mild	Probable
		Admission to hospital	Fever (2)	Mild	Probable
			Gastroenteritis (2)	Moderate	Probable
2	n/a	uk	Asymptomatic (within 1 h of overdose)		
3	68.7	Transfer to psychiatry service	Tiredness (1)	Mild	Possible
			Elevated total cholesterol (4)	Mild	Probable
4	n/a	Admission to hospital for 2 days	Asymptomatic (2)		
5	1.1^a^	No specific measures	Asymptomatic (7 h after overdose)		

h = hours, max. = maximum, n/a = not applicable, uk = unknown.

^a^ maximum licensed dose in combination with cyclosporine used to determine degree of overdose as patient`s usual dose was not known.

### Magnitude of overdose

**Fig 1:**
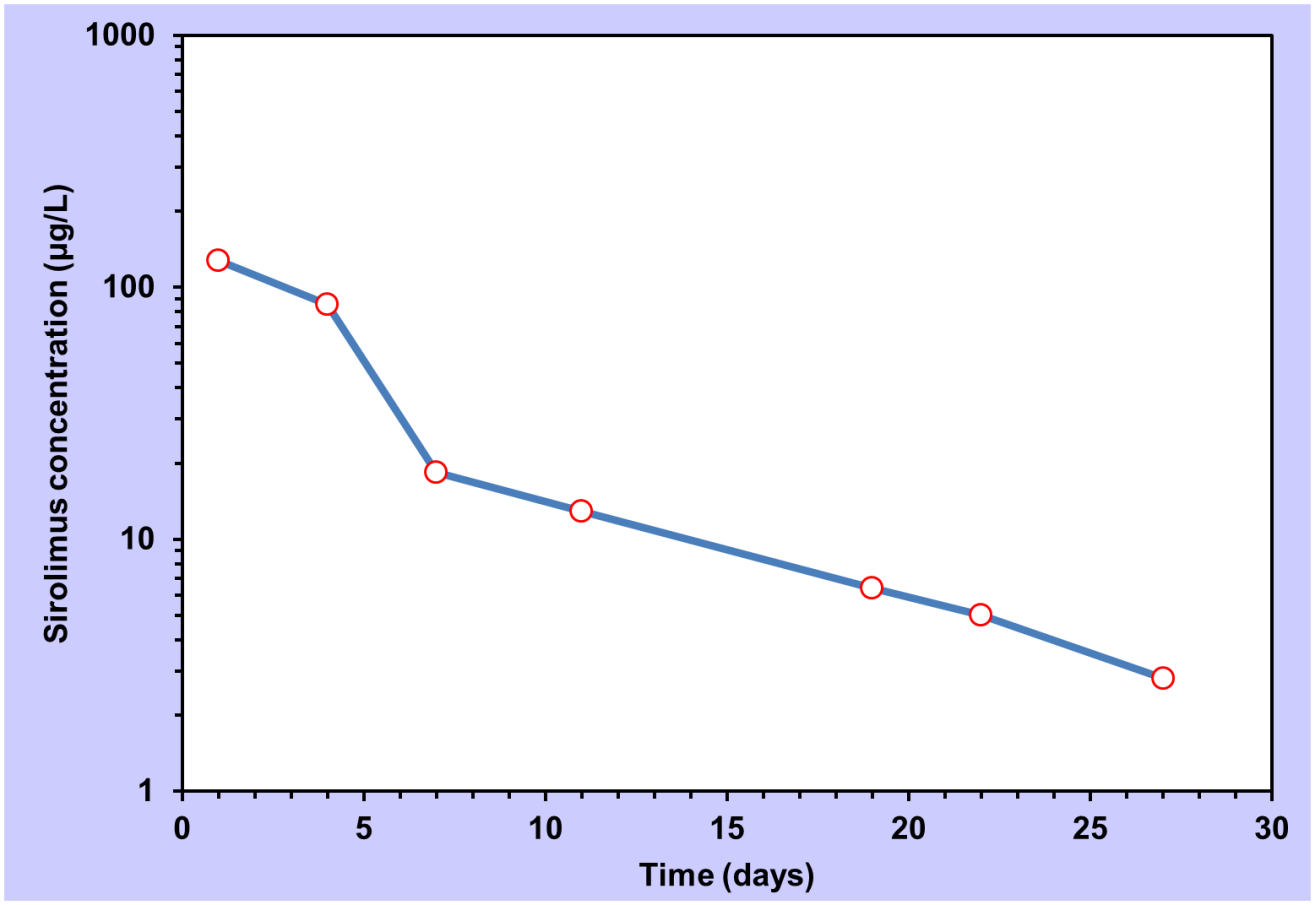
Semi-logarithmic plot of sirolimus concentration vs. time in a single case of overdose with 103 mg (Patient 3). Measurements commenced 24 hours after overdose.

The magnitude of overdose above a patient`s usual maintenance dose or any dose above the maximum licensed dose in treatment-naïve individuals is shown in Table 2. Drug concentration measurements were only available in two cases (patients 1 and 3). Patient 1 had a sirolimus blood concentration of 67.9 μg/L four hours after overdose. Patient 3 underwent serial drug concentration monitoring in order to determine the time for recommencing treatment with sirolimus. The maximum measured whole-blood sirolimus concentration was 127.6 μg/L 24 h after overdose. The concentration-time profile is shown in Fig 1. The patient did not receive sirolimus again until at least 20 days after the overdose. As sirolimus has an oral bioavailability of 15% (when co-administered with cyclosporine) [1], the intravenous application of 1.1 mg in patient 5 was considered approximately equivalent to an oral dose of 6.7 mg (Table 1).

### Signs and symptoms

Signs and symptoms associated with overdose were generally mild (Table 2). There were no life-threatening events. Patient 3, who ingested the largest amount was closely followed but did not show any changes in liver function tests or complete blood count.

### Management

Three patients were hospitalised for at least 24 h. Care was transferred from acute medical to psychiatric in-patient services in one case.

Activated charcoal was administered in patient 1 who presented to the emergency services within four hours of overdose. Patient 3 presented 24 h after overdose, so activated charcoal was not given. Follow-up blood counts and liver function tests were performed in three patients (1, 3 and 4). Serum cholesterol and triglycerides were also measured in patient 3.

### Outcome of overdose

**Fig 2:**
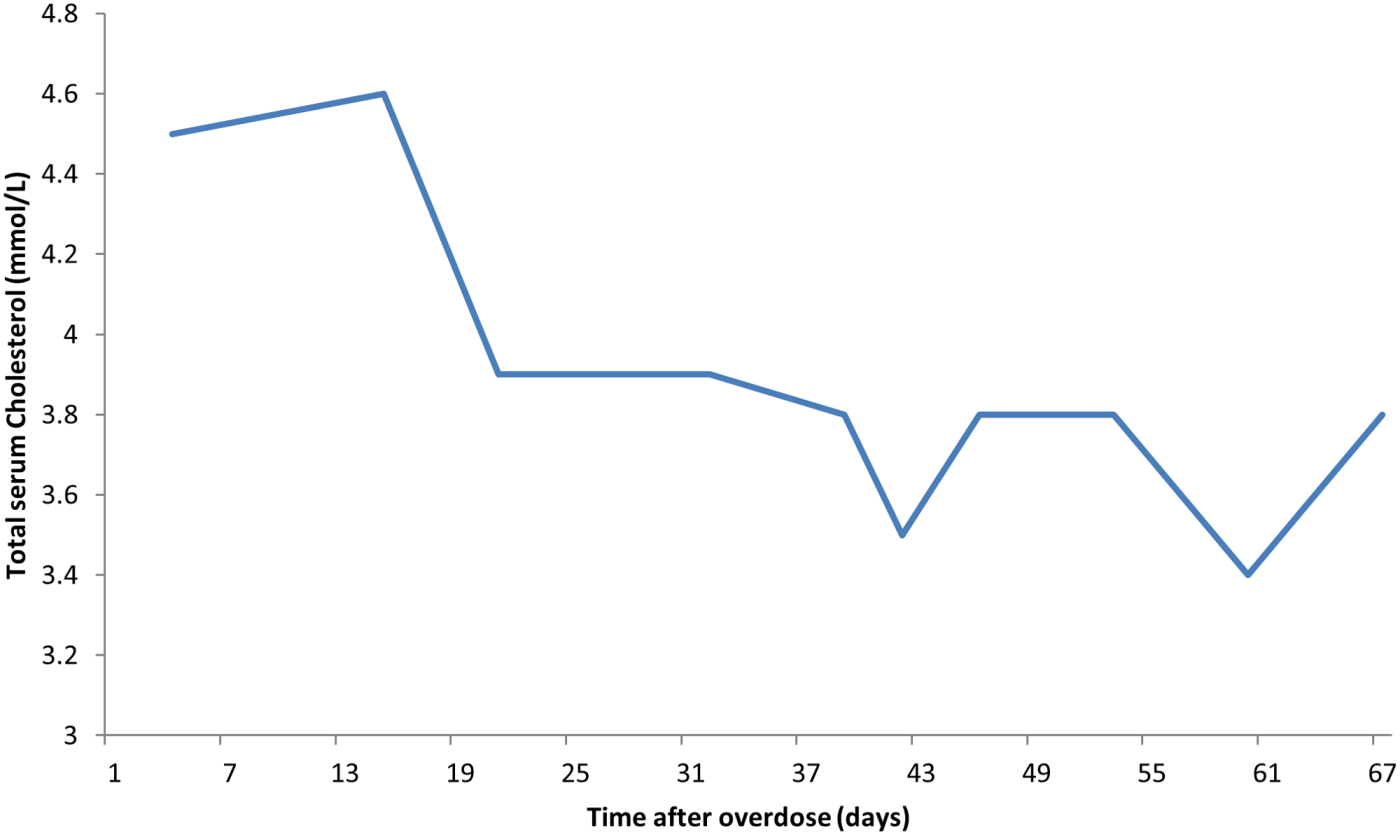
Total serum cholesterol concentration after overdose. The first measurement was taken four days after overdose.

Two sirolimus overdoses were judged to have caused or contributed to symptoms or abnormal clinical findings (Table 2). Patient 1 developed one minor and two moderate symptoms (increased liver enzymes, fever and gastroenteritis) two days after the event. Patient 3 complained of tiredness and showed a spontaneous decline in total cholesterol levels several days after overdose which implied that overdose was associated with an elevation in total cholesterol (Fig 2). Triglyceride concentrations were not above the normal range and did not change significantly from day 4 to day 67 post-overdose (data not shown).

### Pharmacokinetic calculations

Visual inspection of the data presented in Fig 1 indicates at least a two-compartment system. Due to the fact that the first whole blood sirolimus concentration measurement was made 24 h after overdose, it is not possible to determine whether a two- or three-compartment model best fits the data. Pharmacokinetic parameters were determined for the compartments according to the methods given in Section A of the S1 File as shown in Table 3.

**Table 3:** Calculated pharmacokinetic parameters t_1/2_ = half-life, Vd/F = apparent volume of distribution, Cl/F = apparent clearance.

	1st compartment*	2nd compartment
t_1/2_ (h)	51.7	190
Vd/F (L/kg)	0.9	-
Cl/F (L/h/kg)	0.139	-

* The term “compartment” refers to a volume of body fluid into which a drug distributes. Examples of compartments include blood plasma, interstitial fluid and fat tissue.

## Discussion

In this observational case-series analysis of sirolimus overdoses reported to poisons centres within Germany, Austria and Switzerland between 2002 and 2013, we found that acute overdoses appear to be generally well tolerated, with no life-threatening events observed.

### Circumstances and magnitude of overdose

In this series, three of the five overdoses occurred accidentally in children at home and one iatrogenically in the hospital setting. The latter case involved a liquid formulation, which we have observed to be associated with drug-errors involving other immunosuppressants [11]. Therefore, 80% of the overdoses could have been prevented. Doctors who prescribe and pharmacists who dispense sirolimus should therefore be extra vigilant when dealing with unusual formulations and/or administration routes and patients who live with young children. Perhaps repeated instruction to keep medicines out of children’s`reach is required and not just when sirolimus is dispensed for the first time. Due to the fact that absorption and metabolism of sirolimus is so varied between patients, different patients require different doses to achieve the desired therapeutic effect. An overdose was therefore considered to have taken place when this dose was exceeded, and is the reason why we report the multiple of the subject`s usual therapeutic dose in the two patients who were being treated with sirolimus.

### Management

On the basis of its chemical properties, a good adsorption of sirolimus onto activated charcoal is to be expected. Sirolimus has a molecular weight of 914.17 Dalton, which means that it is expected to be readily adsorbed into the 10–1000 Å-sized charcoal pores of current activated charcoal products [15]. Treatment with activated charcoal in cases presenting to health care services within two hours of overdose therefore seems reasonable.

### Signs and symptoms

In one case, elevated alkaline phosphatase, fever and gastroenteritis occurred two days after overdose, and in another total serum cholesterol levels reduced steadily after overdose, the latter implying a concentration-dependent effect of sirolimus as found in other studies [3]. Dyslipidaemia is a well-known dose-dependent adverse effect of mTOR inhibitors that is thought to occur through down-regulation of low-density lipoprotein receptors and inhibition of lipoprotein lipase activity [16]. Elevated triglyceride concentrations are known to be more strongly dose-related than cholesterol concentrations [3] and may have been present early after overdose in patient 3, however no measurements were performed during this time so it is not possible to comment.

It could be speculated that the high serum concentration and long half-life of sirolimus led to significant immunosuppression in patient 1 (a young child) which increased his susceptibility to intercurrent infection.

### Pharmacokinetic profile

Twenty-four hours after taking 103 mg of sirolimus, patient 3`s sirolimus blood concentration was 127.6 μg/L. By way of comparison, 6 healthy, male, adult volunteers had a mean maximum sirolimus concentration (Cmax) of 47.6 μg/L after intake of a single 5 mg/m^2^ dose (approximately 9 mg for an average-sized man) [14].

Several pharmacokinetic studies of sirolimus have shown it to fit a two-compartment model, as we also saw in patient 3 [17, 18]. A pharmacokinetic study of single-dose sirolimus in 16 stable renal transplant recipients (receiving concomitant cyclosporine and prednisone) found a range of half-lives from 39.3 to 86.5 h (compare 51.6 h in patient 3 in the present study) and an 8-fold difference in apparent clearance (0.042 to 0.339 L/h/kg) [18]. The calculated apparent first compartment clearance found in patient 3 of 0.139 L/h/kg falls within this range (Table 3). The apparent volume of distribution for the first (`central`) compartment (0.9 L/kg) is in keeping with sirolimus`protein binding and distribution in blood cells [1] and was also in the range reported by Wu and colleagues in a population pharmacokinetics study (53.4 L) [19]. Although it is not possible to draw conclusions from a single case, the pharmacokinetics of sirolimus in overdose appear to be similar to what is seen in the therapeutic dose range.

### Limitations

Our study has a number of limitations, primarily related to the small sample size. Larger series of sirolimus overdoses have, however, not been published. It is likely that our data did not capture all cases of overdose which occurred in the referral population. Our data are also incomplete, which is the nature of retrospective studies using poison centre data [20], and in one case (patient 5), concomitant cyclosporine intake was assumed. A further limitation is the paucity of data regarding whole blood concentration measurements.

Sirolimus overdose patterns, management and outcomes require further study and clinical toxicologists, nephrologists and treating physicians should be encouraged to actively seek follow-up data on the cases with which they are involved. Further preventive measures are required to reduce the frequency of iatrogenic errors involving liquid formulations and accidental poisoning of children in the home.

## Supporting Information

S1 File Supporting information regarding materials and methods and pharmacokinetic calculations (Section A) and World Health Organisation Uppsala Monitoring Centre (WHO-UMC) causality categories (Table A). (DOC)
