# Supplementary material for: Acute Sirolimus Overdose: A Multicenter Case Series
Source: PLoS One. 2015 May 28;10(5):e0128033. doi: 10.1371/journal.pone.0128033 (PMC4447358; doi:10.1371/journal.pone.0128033)
Supplement: S1 File — (DOC) [file pone.0128033.s001.doc]

**Supporting Information**

**Acute sirolimus overdose: a multicenter case series**

Alessandro Ceschi^1,2^, E. Heistermann^3^, S. Gros^4^, C. Reichert^1^, Hugo Kupferschmidt^1^, Nicholas R. Banner^5,6^, Stephan Krähenbühl^7^, Anne B. Taegtmeyer^7*^

^1^ Swiss Toxicological Information Centre, Associated Institute of the University of Zurich, Zurich, Switzerland

^2^ Department of Clinical Pharmacology and Toxicology, University Hospital Zurich, Zurich, Switzerland

^3^ Berlin Poison Information Centre, Berlin, Germany

^4^ Mainz Poison Control Centre, Mainz, Germany

^5^ The Royal Brompton and Harefield NHS Foundation Trust, Harefield Hospital, Harefield, Middlesex, United Kingdom

^6^ National Heart and Lung Institute and Institute of Cardiovascular Medicine and Research, Imperial College, London, United Kingdom

^7^ Department of Clinical Pharmacology and Toxicology, University and University Hospital Basel, Basel, Switzerland

**Section A**

**Materials and methods**

*Calculation of missing body weight data*

Missing data regarding patient 3`s weight was computed using the mean weight for adult women from a wider population of all calcineurin inhibitor, azathioprine, mycophenolate, 6-mercaptopurine, and sirolimus overdose cases reported to the Swiss Toxicological Information Centre between 1995 and 2011 (n=76) as in our previous publications . The average adult male weight was 69.4 kg; for adult females this figure was 55.8 kg.

*Pharmacokinetic calculations*

Clearance / oral bioavailability (F) was estimated using non-linear-regression analysis for the first compartment as follows:

(1) $Cl/F=\frac{dose}{AUC_{0-\infty}}$

AUC_0- ∞_ was calculated using the trapezoid rule.

The apparent volume of distribution / F for sirolimus was calculated as

(2) $V_{d}/F=\frac{dose}{AUC_{0-\infty}\times k_{e}}$

where k_e_ is the elimination rate constant. K_e_ was calculated as the slope of the elimination phase after semi-logarithmic transformation of the plasma concentration-time curves and was also used to calculate half-life t_1/2_

(3)

**Table A** World Health Organisation Uppsala Monitoring Centre (WHO-UMC) causality categories

| Causality term | Assessment criteria* |
| --- | --- |
| Certain | • Event or laboratory test abnormality, with plausible time relationship to drug intake  • Cannot be explained by disease or other drugs  • Response to withdrawal plausible (pharmacologically, pathologically)  • Event definitive pharmacologically or phenomenologically (i.e. an objective and specific medical disorder or a recognised pharmacological phenomenon)  • Rechallenge satisfactory, if necessary |
| Probable/Likely | • Event or laboratory test abnormality, with reasonable time relationship to drug intake  • Unlikely to be attributed to disease or other drugs  • Response to withdrawal clinically reasonable  • Rechallenge not required |
| Possible | • Event or laboratory test abnormality, with reasonable time relationship to drug intake  • Could also be explained by disease or other drugs  • Information on drug withdrawal may be lacking or unclear |
| Unlikely | • Event or laboratory test abnormality, with a time to drug intake that makes a relationship improbable (but not impossible)  • Disease or other drugs provide plausible explanations |
| Conditional/  Unclassified | • Event or laboratory test abnormality  • More data for proper assessment needed, or  • Additional data under examination |
| Unassessable/  Unclassifiable | • Report suggesting an adverse reaction  • Cannot be judged because information is insufficient or contradictory  • Data cannot be supplemented or verified |

*All points should be reasonably complied with

**References**

1. Ceschi A, Rauber-Luthy C, Kupferschmidt H, Banner NR, Ansari M, et al. (2013) Acute calcineurin inhibitor overdose: analysis of cases reported to a national poison center between 1995 and 2011. Am J Transplant 13: 786-795.
2. Ceschi A, Gregoriano C, Rauber-Luthy C, Kupferschmidt H, Banner NR, et al. (2014) Acute mycophenolate overdose: case series and systematic literature analysis. Expert Opin Drug Saf 13: 525-534.
3. Gregoriano C, Ceschi A, Rauber-Luthy C, Kupferschmidt H, Banner NR, et al. (2014) Acute thiopurine overdose: analysis of reports to a National Poison Centre 1995-2013. PLoS ONE 9: e86390. doi:10.1371/journal.pone.0086390
4. WHO-UMC, *who-****umc****.org/Graphics/24734.pdf* Accessed March 2012.
